# Abnormal fecal microbiota community and functions in patients with hepatitis B liver cirrhosis as revealed by a metagenomic approach

**DOI:** 10.1186/1471-230X-13-175

**Published:** 2013-12-26

**Authors:** Xiao Wei, Xiabei Yan, Dayang Zou, Zhan Yang, Xuesong Wang, Wei Liu, Simiao Wang, Xuelian Li, Juqiang Han, Liuyu Huang, Jing Yuan

**Affiliations:** 1 Institute of Disease Control and Prevention, Academy of Military Medical Sciences, No. 20 Dongda street, Fengtai District, Beijing 100071, China; 2 Institute of Hepatology, Beijing Military General Hospital, No. 5 Dongsishitiao South Gate Warehouse, Dongcheng District, Beijing 100700, China

**Keywords:** Metagenome, Cirrhosis, Microbiota, Metabolism

## Abstract

**Background:**

Assessment and characterization of human colon microbiota is now a major research area in human diseases, including in patients with hepatitis B liver cirrhosis (HBLC).

**Methods:**

We recruited 120 patients with HBLC and 120 healthy controls. The fecal microbial community and functions in the two groups were analyzed using high-throughput Solexa sequencing of the complete metagenomic DNA and bioinformatics methods.

**Results:**

Community and metabolism-wide changes of the fecal microbiota in 20 HBLC patients and 20 healthy controls were observed and compared. A negative correlation was observed between the Child-Turcotte-Pugh scores and *Bacteroidetes* (*P* < 0.01), whereas a positive correlation was observed between the scores and *Enterobacteriaceae* and *Veillonella* (*P* < 0.01). Analysis of the additional 200 fecal microbiota samples demonstrated that these intestinal microbial markers might be useful for distinguishing liver cirrhosis microbiota samples from normal ones. The functional diversity was significantly reduced in the fecal microbiota of cirrhotic patients compared with in the controls. At the module or pathway levels, the fecal microbiota of the HBLC patients showed enrichment in the metabolism of glutathione, gluconeogenesis, branched-chain amino acid, nitrogen, and lipid (*P* < 0.05), whereas there was a decrease in the level of aromatic amino acid, bile acid and cell cycle related metabolism (*P* < 0.05).

**Conclusions:**

Extensive differences in the microbiota community and metabolic potential were detected in the fecal microbiota of cirrhotic patients. The intestinal microbial community may act as an independent organ to regulate the body’s metabolic balance, which may affect the prognosis for HBLC patients.

## Background

The human gut harbors up to 100 trillion microbes, equivalent to 10^11^–10^12^ cells/g colonic content with a biomass of >1 kg [1,2], which encode 100-fold more unique genes than the human genome [3]. Microbial communities in the human gut exhibit high levels of variation; however, they are dominated by members of just two bacterial phyla, *Bacteroidetes* and *Firmicutes*[4]. Obligate anaerobes, such as *Bacteroides*, *Bifidobacterium*, *Bacillus*, and *Peptostreptococcus*, account for 99.9% of the intestinal microbiota, with *Bacteroides* and *Bifidobacterium* accounting for 55% of them [5]. Most of the resident microbes in the gut have a profound influence on human physiology and nutrition [6,7].

Hepatitis B virus (HBV) infection is widespread in China and other Asian countries and chronic HBV infection may develop into cirrhosis after several years. In a previous study, patients with cirrhosis of different etiologies (HBV-related and alcohol-related) had similar fecal microbial communities, which led to the conclusion that the changes observed between the different fecal microbiota were mostly caused by cirrhosis [8]. Intestinal microbiota constitutes a symbiotic ecosystem that keeps homeostatic balance within the human body [6]. However, when cirrhosis develops, portal hypertension promotes gastrointestinal stasis and edema, slowing down of peristalsis, and increased permeability of the gut lining. Moreover, impaired liver function leads directly to decreased detoxification ability and reduced secretion of bile acids [9]. All these factors will disrupt the normal intestinal microbiota equilibrium.

The intestinal microbiota significantly influences the human metabolic phenotype, which involves in a broad co-metabolism between human and microbiota. The anatomy and physiological functions of liver and intestinal microbiota share a close relationship as a result of enterohepatic circulation. The previous study suggested that cirrhosis may be related to the intestinal microbiota structure [8], but how the function and metabolism of intestinal microbiota changed was not addressed. Our understanding of the relationship between human distal gut microbiota and cirrhosis was based mainly on the results of culture-based studies until recent initiatives utilizing a high-throughput sequencing approach [10,11], which made it possible to characterize significant cirrhosis-related alterations in the microbiota community and functions in the present study. Our findings may generate novel perspectives on the progress and prognosis of cirrhosis.

## Methods

### Human fecal sample collection

The Child-Turcotte-Pugh (CTP) scoring system was used to assess the severity of cirrhosis. A total of 240 individuals, including 120 hepatitis B liver cirrhosis (HBLC) patients (40 with CTP score A, 40 with CTP score B, 40 with CTP score C) and 120 healthy individuals, 40–60 years old, with a body mass index (BMI) = 18.5–24.9 kg m^–2^ and without food preferences, were enrolled in this study. Cirrhosis was diagnosed histologically in all patients. None of the patients had comorbid diseases. The control group consisted of 120 normal individuals who visited the People’s Liberation Army 302 Hospital in Beijing for routine health examinations. All healthy individuals had normal liver biochemistry test results with no evidence of hepatic or other diseases. None of the subjects had received antibiotics, probiotics, steroids or other hormones (including oral, intramuscular or intravenous injection) for at least 3 months before sampling. Characteristics of the two groups are given in Table 1 and Additional file 1: Table S1. Patients and normal individuals were each asked to provide a fresh stool sample, which was frozen immediately for DNA extraction. Samples from 20 patients with HBLC (6 with CTP score A, 7 with CTP score B, 7 with CTP score C) and 20 normal individuals were subjected to metagenomic analysis and the other 200 samples were subjected to real-time qPCR analysis. All participants signed an informed consent form prior to entering the study. The study conformed to the ethical guidelines of the 1975 Declaration of Helsinki.

**Table 1:** Characteristics of HBLC patients and controls

**Characteristics**	**HBLC patients (n =120)**			**Controls (n = 120)**
	**CTP-A (n =40)**	**CTP-B (n =40)**	**CTP-C (n =40)**	
HBV-DNA	**Positive**			**Negative**
Age (years)*	46 ± 5	47 ± 8	49 ± 6	48 ± 6
Gender (male/female)	23/17	24/16	26/14	53/67
Body mass index*	22.5 ± 1.3	20.7 ± 1.4	21.4 ± 1.6	23.2 ± 1.1
Total bilirubin (μmol/L)*	19.1.2 ± 4.3	42.7 ± 3.5	79.6 ± 5.8	15.2 ± 4.3
Albumin (g/L)*	42.6 ± 3.7	32.3 ± 2.8	20.5 ± 3.2	44.6 ± 5.2

*Data are expressed as the mean ± SD.

### Metagenomic DNA extraction, DNA library construction and sequencing

A frozen aliquot (200 mg) of each fecal sample was suspended in 250 μL guanidine thiocyanate, 0.1 M Tris (pH 7.5) and 40 μL of 10% thiocyanate. Metagenome extraction was conducted as described previously [12,13]. DNA library preparation was performed according to the manufacturer’s instruction (Illumina). Workflows were designed to perform cluster generation, template hybridization, isothermal amplification, linearization, blocking and denaturation, and hybridization of the sequencing primers. High-throughput Illumina/Solexa sequencing of a 350-bp library for each sample was conducted using an Illumina Genome Analyzer IIx with read lengths of 90 bp. The base-calling pipeline (version Illumina Pipeline-0.3) was used to process the raw fluorescence images and call sequences. High-quality reads were extracted by filtering out low quality reads with ‘N’ bases, adapter contamination, or human DNA contamination from the Illumina raw data.

### Gene catalogue construction

The short read assembler SOAPdenovo 2.20 [12], with the same parameters used to construct the MetaHIT gene catalogue in 2010 [13], was used to assemble the high-quality short reads from the samples. Gene prediction was performed using GeneMark v2.7 [14]. All the predicted genes were aligned pairwise using BLASTN. To construct a non-redundant gene catalogue, sequences that aligned over at least 90% of their length with more than 95% identity (no gaps allowed) were removed as redundancies.

### Bioinformatic analysis

Genes were aligned to the NCBI nr database (e-value < 1e-10, identity > 90%) and taxonomic assignment of the predicted genes was performed using an in-house pipeline as described previously [15]. In this study, samples were clustered using Hellinger distance and then principle component analysis (PCA) was performed to identify the significant features of the intestinal microbes that distinguished patients from healthy individuals. The top five principal components (*P* value in Tracy-Widom test < 0.05 and contribution > 3%) were tested. Using the Wilcoxon rank-sum test method based on the pair-wise comparison matrix, genes with significantly differential abundance between the patients and normal subjects were assessed and subjected to taxonomic assignment at the species level.

Genes were aligned to the eggNOG (v 3.0) and KEGG (release 59.0) databases using BLASTP (e-value ≤1e-5) for functional annotations [16,17]. Each sequence was assigned to a KEGG orthologous group or to an eggNOG orthologous group based on the highest scoring annotated hit(s) that contained at least one high-scoring segment pair (HSP) scoring over 60 bits. For genes with no hits in the eggNOG database, novel gene families were identified based on all-against-all BLASTP results and clustered using the Markov Cluster (MCL) algorithm with an inflation factor of 1.1 and a bit-score cutoff of 60 [15].

### Real-time qPCR

Real-time qPCR was performed using a DNA Engine Opticon 2 apparatus (Bio-Rad, Hercules, CA) with the associated Opticon Monitor software (version 3.0, Bio-Rad). All primer sets used are listed in Table 2[18-20]. Each primer mixture (25μL) contained 12.5μL of SYBR Premix Ex Taq (Takara, Dalian, China) containing MgCl_2_, Tris–HCl, KCl, deoxynucleoside triphosphate, SYBR Green I, and Taq DNA polymerase, 0.5μM primer, and 2μL of the template DNA. Amplifications were performed under the following temperature profiles: one cycle at 95°C for 3 min, 30 cycles of denaturation at 95°C for 30 s, annealing for 40 s, and extension for 45 s, followed by a final elongation step at 72°C for 7 min. Fluorescence was measured after the extension phase of each cycle at an appropriate temperature for 10 s to avoid interference of primer-dimers, secondary structure, or spurious priming. The DNA template concentration was determined by comparison with serially diluting standards (10^1^ to 10^8^ copies of plasmid DNA containing the respective amplicon for each set of primers) running on the same plate. Each reaction was repeated in triplicate on separate run plates.

**Table 2:** Primers used for real-time qPCR

**Target group**	**Primer**	**Sequence (5′-3′)**	**Annealing temperature (°C)**	**Reference**
*Enterobacteriaceae*	Eco1457F	CATTGACGTTACCCGCAGAAGAAGC	63	[18]
	Eco1652R	CTCTACGAGACTCAAGCTTGC		
*Veillonella*	Vei343F	A(C/T)CAACCTGCCCTTCAGA	62	[18]
	Vei343F	CGTCCCGATTAACAGAGCTT		
*Bacteroides*	Bac566F	GGGTTTAAAGGGAGCGTAGG	53	[19]
	Bac692R	CTACACCACGAATTCCGCCT		
*Clostridium*	CI-F	TACCHRAGGAGGAAGCCAC	54	[20]
	CI-R	GTTCTTCCTAATCTCTACGCAT		

### Statistical analysis

A two-tailed Wilcoxon rank-sum test was used to identify the association between microbiota abundance and cirrhosis. Correlation between variables was computed using Spearman rank correlation. The Wilcoxon rank-sum test, Spearman rank correlation and Student t test were conducted using SPSS version 11.0 for Windows (SPSS Inc., Chicago, IL, USA). We applied the “q-value” method proposed previously to estimate the FDR (false discovery rate) instead of a sequential *P*-value rejection method [21]. The statistical hypothesis tests were performed on a large number of features of the gene, KEGG orthologue, and eggNOG orthologue profiles.

## Results

### High-throughput Illumina/Solexa sequencing data

The proportion of high-quality reads in all 40 of the fecal DNA samples was about 91%, and the actual insert sizes ranged from 327–384bp. We obtained an average of 18,852,250 paired-end reads and 8,657,932 single-end reads for each sample, making up a total 1.3 billion high-quality reads that were free of human DNA and adaptor contaminants. We constructed non-redundant gene catalogues that contained 1,603,579 genes from the patient samples (an average of 80,179 genes per sample) and 2,118,215 genes from the normal samples (an average of 105,911 genes per sample).

### Comparison of the microbial structure in the healthy and HBLC samples

**Figure 1:**
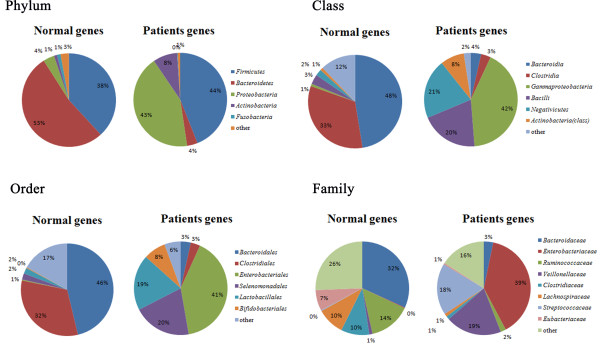
**Composition of fecal microbiota from HBLC patients and healthy individuals.** Data are represented as the average percentage of each individual profile. Fecal microbiota community analysis at the phylum, class, order, family levels demonstrated that the fecal microbiota from the HBLC patients contained a significant absence of *Bacteroidetes* (4% compared with 53% in the microbiota from the controls) and enrichment of *Proteobacteria* (43% compared with 4% in the microbiota from the controls), which included most of the pathogens. The microbiota in the HBLC patients showed a significant enrichment of *Gammaproteobac* (42%), *Negativicutes* (21%), and *Bacilli* (20%) at the class level, *Enterobacteriales* (41%), *Selenomonadales*(20%), and *Lactobacillales* (19%) at the order level, and *Enterobacteriaceae* (39%), *Veillonellaceae* (19%), and *Streptococcaceae* (18%) at the family level.

Analysis on the composition of fecal microbiota (Figure 1) clearly demonstrated that the samples from the cirrhotic patients contained reduced numbers of *Bacteroidetes* and increased numbers of *Proteobacteria* compared with the normal samples. Specifically, our data showed that *Bacteroidetes* made up 53% of the normal fecal microbiota but only 4% of the HBLC fecal microbiota, whereas *Proteobacteria*, which contains most of the opportunistic pathogens, made up only 4% of the normal fecal microbiota but increased to 43% of the HBLC fecal microbiota. These trends observed at the phylum level were also seen at the family level. For example, the *Enterobacteriaceae*, *Veillonellaceae*, and *Streptococcaceae* families made up less than 1% in normal fecal microbiota but were relatively dominant, with proportions ranging from 18–39% in HBLC fecal microbiota, which is consistent with the results of Chen and Zhao [8,22] for intestinal microbiota from cirrhosis patients.

**Figure 2:**
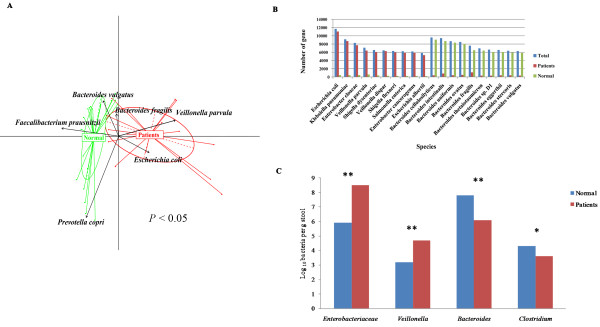
**Comparison of healthy and HBLC microbial structure. (A)** Bacterial species abundance differentiates HBLC patients and healthy individuals. The first five principal components (*P* value in Tracy-Widom test < 0.05 and contribution > 3%) were examined: PC1 = 33.38%, PC2 = 21.81%, PC3 = 12.74%, PC4 = 10.31%, and PC5 = 6.17%. The first two components (PC1 and PC2) are plotted. **(B)** Species annotation of the significantly differential genes. Blue bars represent gene coverage of the significantly differential species, red bars represent gene coverage of the significantly differential species in the HBLC samples, and green bars represent gene coverage of the significantly differential species in the control samples. **(C)** Bacterial groups quantified using real-time qPCR. Blue bars represent the control samples, and red bars represent the HBLC samples. The Student t test was used to evaluate the statistical difference between the two groups. *P < 0.05; ** *P* < 0.01.

To further investigate the differences in fecal microbiota between the HBLC and normal samples, PCA was employed. We found that the first and second principal components clearly separated the normal and HBLC fecal microbiota structures (Figure 2A). Some of the species with significantly different abundance between the two groups were *Escherichia coli*, *Veillonella dispar*, *Veillonella parvula*, which were significantly enriched, and *Bacteroides* species, which were reduced, in the HBLC samples compared with the normal samples.

To find which species were significantly absent or enriched in patients, we used the Wilcoxon rank-sum test method to analyze the genes that were in significantly differential abundance between the HBLC and normal fecal microbiota. We found that 49,442 genes and 72,584 genes were significantly enriched (*P* < 0.01) in patients and normal samples, respectively. Of these, a total of 103,099 genes could be matched to known species with a large ratio of coverage (Figure 2B). We found that the fecal microbiota from the cirrhotic patients contained a remarkable absence of *Bacteroides cellulosilyticus*, *Bacteroides intestinalis*, *Bacteroides uniformis*, *Bacteroides ovatus*, *Bacteroides_fragilis*, *Bacteroides thetaitaomicron*, *Bacteroides* sp.D1, *Bacteroides eggerthii*, *Bacteroides stercoris*, and *Bacteroides vulgates*, all of which belong to the *Bacteroides* genus (*P* < 0.01) and were negatively correlated with the CTP scores (R < −0.7). In contrast, the fecal microbiota from the cirrhotic patients contained high abundances of *Escherichia coli*, *Klebsiella pneumonia*, *Enterobacter cloaca*, *Veillonella_parvula*, *Shigella dysenteriae*, *Veillonella dispar*, *Shigella flexneri*, *Salmonella enteric*, *Enterobacter cancerogenus*, and *Escherichia albertii*, all of which belong to the *Veillonella* genus or *Enterobacteriaceae* family (*P* < 0.01) and were positively correlated with the CTP scores (R > 0.7).

A comparison of the dominant and subdominant bacteria genera between the HBLC and normal fecal microbiota was carried out by real-time qPCR (Figure 2C). Compared with the normal samples, *Enterobacteriaceae* and *Veillonella* were significantly increased, and *Bacteroides* and *Clostridium* were significantly decreased in the HBLC samples, confirming the results obtained from the high-throughput Illumina/Solexa sequencing.

### Functional characterization of HBLC fecal microbiota

**Figure 3:**
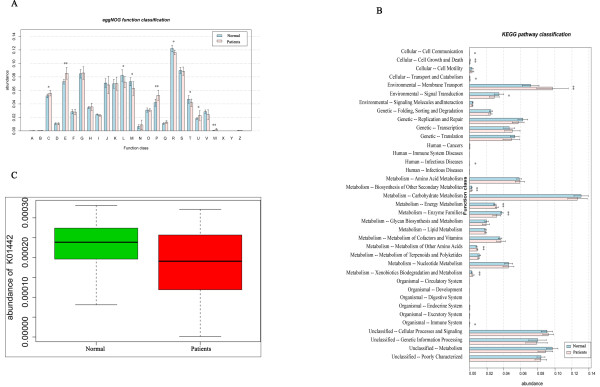
**Function and metabolism analysis of fecal microbiota from HBLC patients and control samples.** Blue bars represent the control samples, and red bars represent the HBLC samples. The Student t test was used to evaluate the statistical difference between the two groups. *P < 0.05; ** *P* < 0.01 **(A)** Genes annotated using the eggNOG database. The significantly differential genes were annotated as: E, amino acid transport and metabolism; P, inorganic ion transport and metabolism; Q, secondary metabolites biosynthesis, transport and catabolism; and W, extracellular structures. **(B)** Genes annotated using the KEGG database. The significantly differential genes were annotated with pathways: Cellular, cell growth and death; Environmental, membrane transport; Environmental, signal transduction; Metabolism, biosynthesis of other secondary metabolites; Metabolism, energy metabolism; Metabolism, enzyme families; Metabolism, metabolism of other amino acids; and Metabolism, xenobiotics biodegradation and metabolism. **(C)** Relative abundance of genes annotated as BSH related to primary and secondary bile acid biosynthesis [KEGG: K01442]. Green bars represent the control samples, and red bars represent the HBLC samples. The Student t test was used to evaluate statistical difference between the two groups, *P* = 0.013.

A functional analysis of our data revealed functions that were enriched or decreased in the HBLC fecal microbiota compared with the normal fecal microbiota (Figure 3A, B). At the highest hierarchical levels, fecal microbiota from HBLC patients revealed an enrichment in amino acid transport and metabolism (*P* < 0.01), secondary metabolites biosynthesis, transport and catabolism (*P* = 0.005), inorganic ion transport and metabolism (*P* = 0.001), extracellular structures (*P* = 0.001), energy production and conversion (*P* = 0.014), and intracellular trafficking and secretion (*P* = 0.019), and a decrease in cell wall/membrane biogenesis (*P* = 0.047), signal transduction metabolism (*P* = 0.02), replication, recombination and repair (*P* = 0.018), and general function prediction only (*P* = 0.012). Interestingly, for most of these strikingly different functions, the relative abundance of genes in each category was statistically lower in the HBLC microbiota samples compared with their abundances in the normal microbiota samples, indicating a reduction in functional diversity in the HBLC microbiota.

Bile salt hydrolases (BSHs) are members of the choloylglycine hydrolase family (EC 3.5.1.24) and are important in bile acid metabolism. BSHs have been isolated and/or characterized from several species of intestinal bacteria [23]. We found that genes annotated as BSH related to primary and secondary bile acid biosynthesis [KEGG:K01442] were in much higher abundance in the normal microbiota than in the HBLC microbiota (*P* = 0.013) (Figure 3C).

Human gut bacteria are expected to encounter a broad spectrum of carbohydrate substrates. The fecal microbiota from the cirrhotic patients showed enhanced metabolic ability for carbohydrate transportation because of the high abundance of genes involved in phosphotransferase systems (*P* = 0.006) and ABC transporters (*P* = 0.004). Furthermore, the HBLC microbiota showed an enhanced ability to transform non-carbohydrate carbon substrates into glucose because of the high abundance of genes involved in the metabolism of gluconeogenesis (GNG) and pyruvate (pyruvate is one of the main gluconeogenic precursors) (*P* < 0.05).

In the lipid metabolism KEGG pathway, the fecal microbiota from the HBLC patients was significantly enriched in genes associated with fatty acid metabolism (R = 0.84, *P* = 0.025), unsaturated fatty acids biosynthesis (R = 0.59, *P* = 0.018), glycerophospholipid (R = 0.82, *P* = 0.004), alpha-linolenic acid (R = 0.77, *P* = 0.007), and butanoate (R = 0.78, *P* = 0.011), which may provide additional energy resources in these patients.

In the amino acid metabolism pathway, the fecal microbiota from HBLC patients was significantly enriched in genes associated with the metabolic ability of branched-chain amino acid (BCAA) involved in the valine, leucine and isoleucine biosynthesis (*P* = 0.030) and degration (*P* = 0.002). Conversely, genes associated with the metabolism of aromatic amino acids, including tyrosine (*P* = 0.0007), phenylalanine (*P* = 0.011), were less abundant in the microbiota from HBLC patients than in the microbiota from the healthy controls.

In addition, the fecal microbiota of HBLC patients were enriched for glutathione metabolism (*P* = 0.008), glutathione synthase (EC 6.3.2.3) and glutathione reductase (NADPH) (EC 1.8.1.7) (*P* < 0.01).

In brief, our data showed that the fecal microbiota structure, as well as a variety of functions were different in HBLC patients compared with in the normal controls, suggesting that the microbiota had changed to adjust to the cirrhosis-related intestinal microenvironment.

## Discussion

The human gut harbors a vast ensemble of microbes and has the highest recorded density of any microbial habitat in nature [2]. Genes of intestinal flora are considered as a second genome, which can affect the health status of humans [13]. The reduced intestinal blood perfusion, mesenteric ischemia, and decreased bowel movement caused by cirrhosis appears to have changed the normal microenvironment that is suitable for beneficial populations *Bacteroides* and *Clostridium*, thereby allowing opportunistic pathogens like *Enterobacteriaceae* and *Veillonella* to invade and colonize. *Veillonella* can hydrolyze conjugated bile salts and promote the impairment of micelle formation or cirrhosis. The abnormal intestinal microbiota structure that we detected in HBLC patients may negatively affect their prognosis.

Our data revealed striking functional differences between the patients’ fecal microbiota and the normal fecal microbiota. The fecal microbiota from the cirrhotic patents contained genes that are important for toxins depredation and nutrient absorption. For nearly every major function, the relative abundance of the associated genes was statistically lower in the patients compared with the controls. Furthermore, the microbiota from the cirrhotic patients contained a marked enhancement of genes related to material transport and metabolism and an absence of genes for metabolism related to cell cycle. Therefore, we inferred that the microbiota from HBLC patients harbored more fastidious bacteria that required more nutrients in the external environment for survival and growth.

Glutathiones, with broad-spectrum detoxification activity, are involved in biotransformations that change the harmful toxins in the body into harmless substances that can be excreted. Most of the intestinal anaerobic bacteria do not have a glutathione metabolic pathway, so we inferred that the microbiota genes that were enriched for glutathione metabolism in the HBLC patients might have arisen as a result of the decrease in the abundance of *Bacteroidetes*. Large doses of administered drugs, impaired liver function, and increased toxins from opportunistic pathogens or drug accumulation in a patients’ intestine, may stimulate a compensatory mechanism in the microbiota to adjust their metabolic structure to degrade the harmful toxins.

Strong correlations of interdependence, mutual competition, and mutual inhibition are known to occur between bile acids and the intestinal microbiota community. Conjugated bile acids released into the intestine are hydrolyzed by BSHs, which are secreted mainly by *Bacteroides* and *Clostridium*, to form deconjugated bile acids [24,25]. These bacteria may be crucial for the 7α-dehydroxylation of free-form primary bile acids, so that the secondary bile acids can be combined with insoluble fiber flocculates or absorbed through the membranes of cells lining the colon, thereby reducing the bile acid concentration in the intestine. The dissociation role catalyzed by BSH provides carbon, nitrogen, and sulfur sources and energy for *Bacteroides* and *Clostridium*[24,25]. Our data showed that the BSH level was significantly reduced in fecal microbiota from the HBLC patients, which was concomitant with the reduced abundance of *Bacteroides* and *Clostridium*.

The fecal microbiota of the patients showed the ability to compensate for the impaired liver functions in terms of energy and nutrient metabolism. In healthy individuals, GNG takes place mainly in the liver to keep blood glucose levels from dropping too low. The decreased content of hepatic glycogen caused by impaired liver function could stimulate GNG metabolism in HBLC patients. Our data suggested that the fecal microbiota of HBLC patients also showed enhanced GNG, resulting in the generation of glucose from non-carbohydrate carbon substrates such as pyruvate, to maintain the body’s energy supply. Cirrhotic patients, who use lipids instead of carbohydrate as the main energy source, had decreased level of total fatty acid and unsaturated fatty acids in their plasma. The fecal microbiota in the HBLC patients seemed to play a compensatory role by enhancing unsaturated fatty acids synthesis, fatty acid metabolism, and glycerophospholipid metabolism, which may provide energy resources for the patients. HBLC patients have weak fitness and as a result the peripheral tissues need to increase their consumption of BCAA. Interestingly, the fecal microbiota in HBLC patients also significantly enhanced the biosynthesis of valine, leucine and isoleucine, which are all BCAA, to partially supplement the need for BCAAs.

## Conclusions

Intestinal microbiota acts as a fundamental system in humans, regulating the body’s metabolic balance by gene adjustment and microbe restructuring, which are important in disease prognosis. Further comparative investigations into the interactions between human and microbial communities, including whether a core set of genes or functional biomarkers are associated with the microbiota of HBLC patients versus the microbiota of healthy individuals. Genetic or behavioral traits of the host that can reshape the microbiota communities also need to be investigated further.

## Abbreviations

BCAA: Branched-chain amino acid; BMI: Body mass index; BSH: Bile salt hydrolases; COG: Clusters of orthologous groups of proteins; EC: Enzyme commission; GNG: Gluconeogenesis; HBLC: Hepatitis B liver cirrhosis; HBV: Hepatitis B virus; KEGG: Kyoto encyclopedia of genes and genomes; NADPH: Glutathione reductase; PCA: Principle component analysis; PTS: Phosphotransferase system; SOAP: Short oligonucleotide alignment program.

## Competing interests

The authors declare that they have no competing interests.

## Authors’ contributions

XW designed the study, performed most of the bioinformatic analysis and drafted the manuscript. XY participated in the design of the study and performed the statistical analysis. DZ participated in the sequence alignment. ZY participated in sampling and extracted the metagenomic DNA. XW conceived of the study. WL participated in bioinformatic analysis and helped to draft the manuscript. SW carried out the qRT-PCR experiment. XL participated in the bioinformatic analysis. JH participated in drafting the manuscript and revising it. LH participated in conceiving the study. JY helped to draft the manuscript and approved the final version to be published. All authors read and approved the final manuscript.

## Pre-publication history

The pre-publication history for this paper can be accessed here:

http://www.biomedcentral.com/1471-230X/13/175/prepub

## Supplementary Material

Additional file 1: Table S1 Number of patients with different HBV-DNA copies. Click here for file
