# Supplementary material for: Abnormal fecal microbiota community and functions in patients with hepatitis B liver cirrhosis as revealed by a metagenomic approach
Source: BMC Gastroenterol. 2013 Dec 26;13:175. doi: 10.1186/1471-230X-13-175 (PMC3878425; doi:10.1186/1471-230X-13-175)
Supplement: Additional file 1: Table S1 — Number of patients with different HBV-DNA copies. [file 1471-230X-13-175-S1.docx]

**Additional file 1 Table S1 Number of patients with different HBV-DNA copies**

| HBV-DNA (copies/ml) | Number of HBLC patients* | | |
| --- | --- | --- | --- |
|  | CTP-A^#^ | CTP-B^#^ | CTP-C^#^ |
| <10^3^ | 15 | 14 | 14 |
| 10^3^-10^4^ | 16 | 18 | 14 |
| 10^4^-10^5^ | 8 | 5 | 9 |
| 10^5^-10^6^ | 0 | 2 | 3 |
| 10^6^-10^7^ | 1 | 1 | 0 |
| Total | 40 | 40 | 40 |

*Total number of HBLC patients is 120.

^#^Total number of patients with CTP score of A is 40, with CTP score of B is 40, and with CTP score of C is 40.

The correlation between viral load and microbiota community and functions were analyzed.The correlation index between viral load and *Bacteroides* (R = -0.08), *Veillonella* (R = -0.17), and *Escherichia* (R = -0.16) were not statistically significant. Also, the correlation index between viral load and glutathione metabolism (map00480, R = 0.02), propanoate metabolism (map00640, R = 0.01), valine, leucine and isoleucine degradation (map00280, R = 0.10) and synthesis (map00290, R = -0.07) were not statistically significant. In brief, the data showed that there is no correlation between viral load and microbiota community and functions.
